# Rest-activity profiles among U.S. adults in a nationally representative sample: a functional principal component analysis

**DOI:** 10.1186/s12966-022-01274-4

**Published:** 2022-03-24

**Authors:** Qian Xiao, Jiachen Lu, Jamie M. Zeitzer, Charles E. Matthews, Pedro F. Saint-Maurice, Cici Bauer

**Affiliations:** 1 Department of Epidemiology, Human Genetics and Environmental Health, School of Public Health, the University of Texas Health Science Center at Houston, 1200 Pressler St., TX Houston, USA; 2 Department of Biostatistics and Data Science, The University of Texas Health Science Center at Houston, 1200 Pressler St., TX Houston, USA; 3 Department of Psychiatry and Behavioral Sciences, Stanford University, Stanford, CA USA; 4 Metabolic Epidemiology Branch, Division of Cancer Epidemiology and Genetics, National Cancer Institute, Bethesda, MD USA

**Keywords:** Rest-activity cycle, Physical activity, Sedentary behavior, Sleep, Circadian rhythms, Population differences, Self-rated health, Examine whether and how variations in rest-activity patterns may contribute to health disparities

## Abstract

**Background:**

The 24-h rest and activity behaviors (i.e., physical activity, sedentary behaviors and sleep) are fundamental human behaviors essential to health and well-being. Functional principal component analysis (fPCA) is a flexible approach for characterizing rest-activity rhythms and does not rely on a priori assumptions about the activity shape. The objective of our study is to apply fPCA to a nationally representative sample of American adults to characterize variations in the 24-h rest-activity pattern, determine how the pattern differs according to demographic, socioeconomic and work characteristics, and examine its associations with general health status.

**Methods:**

The current analysis used data from adults 25 or older in the National Health and Nutrition Examination Survey (NHANES, 2011–2014). Using 7-day 24-h actigraphy recordings, we applied fPCA to derive profiles for overall, weekday and weekend rest-activity patterns. We examined the association between each rest-activity profile in relation to age, gender, race/ethnicity, education, income and working status using multiple linear regression. We also used multiple logistic regression to determine the relationship between each rest-activity profile and the likelihood of reporting poor or fair health.

**Results:**

We identified four distinct profiles (i.e., high amplitude, early rise, prolonged activity window, biphasic pattern) that together accounted for 86.8% of total variation in the study sample. We identified numerous associations between each rest-activity profile and multiple sociodemographic characteristics. We also found evidence suggesting the associations differed between weekdays and weekends. Finally, we reported that the rest-activity profiles were associated with self-rated health.

**Conclusions:**

Our study provided evidence suggesting that rest-activity patterns in human populations are shaped by multiple demographic, socioeconomic and work factors, and are correlated with health status.

**Supplementary Information:**

The online version contains supplementary material available at 10.1186/s12966-022-01274-4.

## Introduction

Physical activity, sedentary behaviors and sleep are fundamental human movement behaviors organized in a 24-h rhythmic cycle. These behaviors are orchestrated by the internal circadian timing system, and influenced by common environmental exposures (e.g., light, daily schedules and social interactions) [1]. The conventional approach to study diurnal movement behaviors focuses on measures of individual components such as physical activity intensity and volume, duration of sitting, and sleep duration and efficiency. However, However, there’s been little focus on the timing and rhythmic profiles of these behaviors and movement over the 24-h day. The highly interconnected nature of these behaviors requires an integrated and holistic approach to study the overall patterns of the 24-h rest-activity cycle [2].

Multi-day accelerometry collects rich and complex data for investigating movement behaviors, and its increasing popularity in population studies has presented as both a challenge and opportunity for characterizing 24-h rest-activity rhythms. Using accelerometry data, previous studies have derived both parametric (e.g., amplitude, acrophase and mesor from cosinor-based models) [3] and nonparametric (e.g., interdaily stability, intradaily variability and relative amplitude) [4, 5] metrics to characterize the rest-activity cycle and reported associations with various health outcomes, including diabetes [6], cognitive decline [7–9], inflammation [10], and mortality [11]. Although these investigations produced crucial evidence linking rest-activity characteristics with health and diseases, their characterization of rest-activity has several methodological limitations: 1) most metrics only capture specific aspects of the rest-activity cycle and fail to assess overall rhythmicity; 2) parametric approaches lack the flexibility in evaluating profiles that deviate from the assumed shape. An alternative approach to overcome these limitations is the functional principal component analysis (fPCA), which applies flexible algorithms to fit activity data with no a priori assumptions and is able to identify overall rest-activity profiles. Several recent studies demonstrated the utility of the fPCA approach, and used it to link rest-activity profiles with multiple health outcomes, such as apathy in individuals with Alzheimer disease [12], mood states among bipolar patients [13], and sleep, cognitive decline and mortality in healthy aging men [14].

Characterizing rest-activity patterns in the general population and by demographic, socioeconomic and work characteristics provides key information to identify subgroups with sub-optimal rest-activity patterns, and shed light on individual and environmental factors shaping the rest-activity behaviors. To date, few studies examined demographic and lifestyle correlates with rest-activity patterns [15–17], and most used small samples with limited generalizability. The National Health and Nutrition Examination Survey (NHANES) is a biennial national survey which includes a large sample representative of the US population. In the 2011–2012 and 2013–2014 cycles, the NHANES included the Physical Activity Monitor module, which collected 24-h actigraphy data for 7 consecutive days. This represents a substantial improvement over the first NHANES accelerometry protocol in 2003–20,066 where accelerometry monitoring was limited to wake time and provides an ideal opportunity to study 24-h rest-activity patterns in sociodemographically diverse populations. In the current study, we applied fPCA to characterize rest-activity patterns in NHANES, and studies their relationships to demographic attributes, socioeconomic status (SES) and work status. To better evaluate the public health implications of the variations in rest-activity patterns across different subpopulations, we also examined the relationship between rest-activity profiles and self-rated health, an indicator of overall health status [18].

## Methods

### Study population

NHANES is a biennial cross-sectional survey designed to assess the health and nutritional status of non-institutionalized population in the United States and conducted by the National Center for Health Statistics at the Center for Disease Control and Prevention [19]. The NHANES uses a four-stage probability sampling design and oversamples racial/ethnic minority groups (Hispanic, non-Hispanic Black and since 2011, non-Hispanic Asian), older adults and low income populations to improve the reliability and precision of estimates among these subgroups [19]. This current analysis used data from 2011–2012 and 2013–2014 cycles, in which 24-h actigraphy data were collected, along with sociodemographic characteristics and health status.

### Physical activity measurement and fPCA-based rest-activity profiles

Details of the physical activity monitor protocol have been published online [20]. Briefly, participants wore an ActiGraph GT3X + (Pensacola, Florida) on the wrist of the non-dominant hand for seven consecutive days except during showering and water-based activities [21]. Valid measurements after data quality control were categorized as wake wear, sleep wear or non-wear using an open-source algorithm [21, 22]. We used the minute-by-minute triaxial acceleration measures, Monitor-Independent Movement Summary units (MIMS), and coded any invalid measure or non-wear time as missing.

We used R packages fda to perform the fPCA [23] to derive rest-activity profiles for each participant, for the overall activity and also stratified by weekdays/weekends. We first calculated the mean activity value for each 5-min epochs (*N* = 288/day) across all available days and fitted a curve with Fourier bases to create a single “smoothed” activity pattern for the 24-h period. We then performed standard PCA on this activity pattern, and the eigenvectors from the fPCA were extracted for the top components, each representing a distinct feature, or profile, of the rest-activity cycle. For each PCA component, the associated eigenvalue quantifies to what degree the participent’s 24-h rest-activity pattern reflects the specific rest-activity profile characterized by this PCA component, and was used in subsequent association studies. The number of principle components was determined by a scree plot. Our code for the fPCA and a sample dataset are available at https://github.com/cicibauer/fpca_NHANES.

### Participant characteristics

Trained interviewers gathered sociodemographic information from the participant family and sample person, and we focused on the following: 1) age (25–29, 30–39, 40–49, 50–59, 60–69, and 70 +); 2) gender (male, female); 3) race/ethnicity (non-Hispanic White, non-Hispanic Black, Mexican American, Other Hispanic, Non-Hispanic Asian, and others); 4) education (less than high school, high school graduate, some college, college graduate or higher); 5) household income (< $20 k, $20 k-$44.9 k, $45 k-$74.9 k, ≥ $75 k); 6) work status in the past week (did not work, worked for < 40 h, worked 40 h or more). Study participants also reported self-rated health status as excellent, very good, good, fair or poor, from which we created a dichotomous variable indicating whether a participant reported poor or fair health. Distribution of sociodemographic variables and self-rated health are presented in Supplementary Table 1. Analytic sample.

A total of 19,931 participants were in NHANES 2011–2014. We focused on adults 25 or older (*N* = 10,275) because socioeconomic indicators assessed among younger adults in the early 20 s may not accurately reflect their lifelong SES. We excluded those missing education (*N* = 11), income (*N* = 894) and work status (*N* = 6) (none missing age, gender or race/ethnicity). We further excluded those with no actigraphy data (*N* = 1,378) or missing one or more 5-min epochs across all seven days (*N* = 329). The overall analysis included 7,657 participants. The stratified analysis investigating the weekday-weekend differences had 7,041 participants with both weekday and weekend rest-activity profiles (detailed below). The analysis on self-rated health excluded participants with missing self-reported health status (*N* = 528).

### Statistical analysis

The association between participant characteristics and rest-activity profiles was determined by multiple linear regression, where the PCA values were the outcome variable, and participant characteristics as the explanatory variables. The reference group for each explanatory variable was chosen as follows: For age, we chose the youngest group (25–29) because it was presumed to be the healthiest. For gender and race/ethnicity, we chose men and NH White because they had the larger/largest sample size. For all SES and work variables, we chose the group with presumably the highest SES (i.e., college graduate for education, ≥ $75 k for income, and worked 40 h or more for work status). To determine the association between rest-activity profiles and self-rated health, we first divided the eigenvalue for each component, or rest-activity profile, into quintiles and then used multiple logistic regression to calculate the odds ratio (OR) and 95% confidence intervals (CI) for reporting fair or poor health comparing Q1-Q4 to Q5 (reference group), adjusting for age, gender, race/ethnicity, education, income and work status. We performed analyses for overall activity and for weekday- and weekend-specific patterns separately. All analyses accounted for the complex sample design by including NHANES full sample weights (calculated as ½ * WTMEC2YR). We performed fPCA using R package fda (version 5.1.9) and the rest of the analyses were performed in SAS (version 9.4, Cary NC).

## Results

**Fig. 1:**
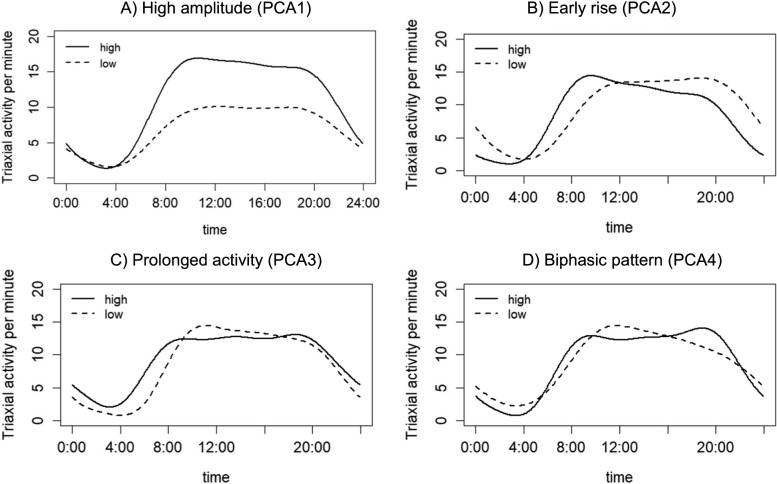
Rest-activity profiles of 24-hour actigraphy data from adults in the National Health and Nutrition Examination Survey (2011-2014). Each panel depicts the mean 24-hour activity patterns for participants with high (solid line) and low (dotted) eigenvalues of the first four components derived from the functional principal component analysis (PCA): **A** The first component (50.2% variance), with higher eigenvalues representing a higher amplitude; **B** the second component (21.6% variance), with higher eigenvalues representing earlier rise time; **C** the third component (9.4% variance), with higher eigenvalues representing a more prolonged daytime activity window; **D** the forth component (5.6% variance), with higher eigenvalues representing a more pronounce biphasic pattern characterized by a mid-day dip in activity

Figure 1 presents the average rest-activity profiles with a high vs low eigenvalue (above vs below median) for each of the top four PCA components, which together explained 86.8% of the total variance in the rest-activity profiles. The four top components are: 1) The high amplitude activity profile (PCA1): A higher value for this component suggested a higher level of physical activity throughout the day (Fig. 1A). 2) The early rise profile (PCA2): A higher values for this component suggested an earlier timing for the rising phase of the daytime active period (Fig. 1B). 3) The prolonged activity profile (PCA3): A higher value for this component suggested a longer daytime active period (Fig. 1C). 4) The biphasic activity profile (PCA4): A higher value suggested a pattern with two activity peaks in the morning and the evening and a dip in activity level in the middle of the day (Fig. 1D). The activity profiles for weekdays and weekends were largely similar to those in the overall analysis (Supplementary Figs. 1 and 2), showing mostly moderate-to-high correlation coefficients among each set of the top four components for overall, weekday and weekend fPCA results (Supplementary Table 2). The correlation between weekday and weekend profiles was higher for PCA1 and PCA2 (correlation coefficients, 0.72 and 0.65, respectively). In contrast, the weekday-weekend correlation for PCA4 was low (0.14) and the weekend component exhibited a more prominent later peak than a biphasic pattern, suggesting a distinct profile.

**Fig. 2:**
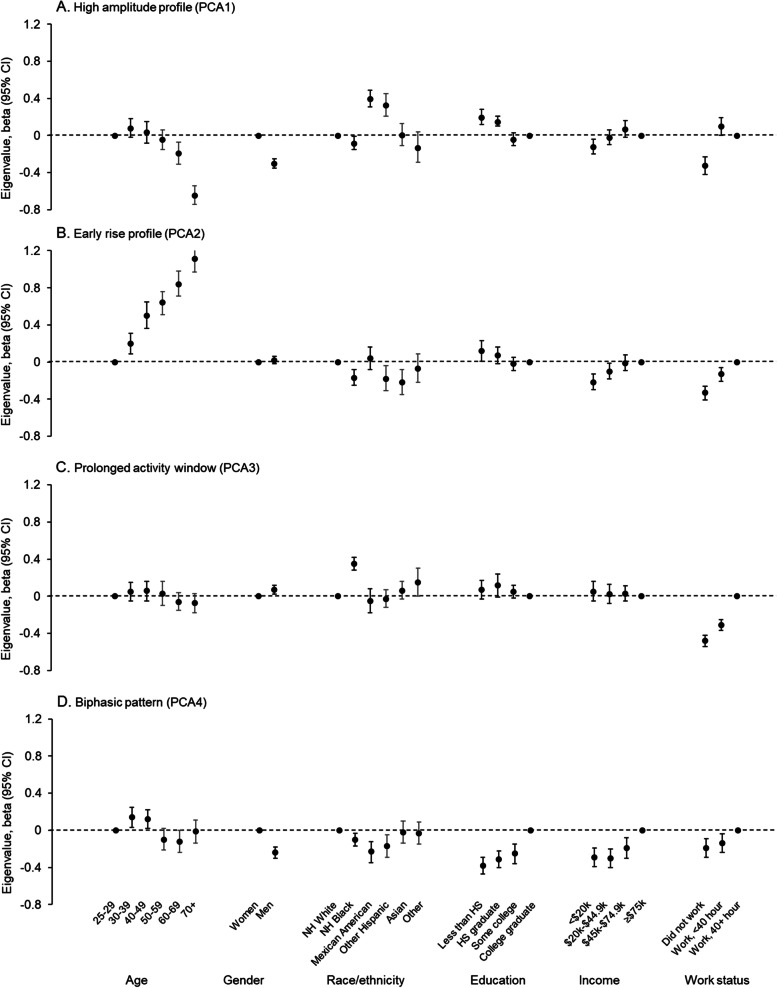
Associations between study characteristics and rest-activity profiles in adults in the National Health and Nutrition Examination Survey (2011-2014). Multiple linear regression models included all study characteristics and their categories simultaneously. Abbreviations: CI, confidence interval; HS, high school; NH, non-Hispanic; PCA1-4, principal component analysis component 1-4

Each rest-activity profile was associated with multiple participant characteristics (Fig. 2 and Table 1). A higher eigenvalue for the high amplitude profile (PCA1) was observed among Mexican American and the other Hispanic ethnic groups, and those who had lower education, and/or reported working for < 40 h during the previous week, while a lower amplitude was observed among older adults, men, and participants with household income < $20 k and/or reporting not working, compared to the relevant reference groups (Fig. 2A). A higher eigenvalue for the early rise profile (PCA2) was strongly associated with older age (Fig. 2B), and lower education levels. In contrast, a lower value for this profile was observed among participants who were non-Hispanic Black, Asians and in the other Hispanic group, had a household income < $45 k and reported not working or working for < 40 h. The prolonged activity window profile (PCA3) was most strongly associated with work status, with those reporting not working or working for < 40 h showing a higher eigenvalue for this profile (Fig. 2C). Moreover, non-Hispanic Black participants had a significantly increase in the eigenvalue for this profile when compared to their non-Hispanic White counterparts. A lower eigenvalue for the biphasic activity profile was associated with lower education and income levels (Fig. 2D). In addition, men, non-Hispanic Black and Hispanic groups and those who reported not working or working for < 40 h also had a lower eigenvalue for the biphasic pattern profile.

**Table 1:** Associations ^a^ between study characteristics and rest-activity profiles in adults in the National Health and Nutrition Examination Survey (2011-2014)

	**PCA1 - high amplitude**		**PCA2 - early rise**		**PCA3 - prolonged activity window**		**PCA4 – biphasic pattern**	
	**Median (IQR)**	**β (95% CI)**	**Median (IQR)**	**β (95% CI)**	**Median (IQR)**	**β (95% CI)**	**Median (IQR)**	**β (95% CI)**
**Age**								
25-29	0.27 (-0.39, 0.86)	ref	-0.43 (-1.18, 0.15)	ref	-0.14 (-0.66, 0.45)	ref	0.11 (-0.55, 0.81)	ref
30-39	0.27 (-0.36, 0.93)	0.08 (-0.02, 0.18)	-0.20 (-0.85, 0.38)	0.20 (0.09, 0.31)	-0.08 (-0.56, 0.51)	0.05 (-0.05, 0.15)	0.32 (-0.41, 0.94)	0.14 (0.03, 0.25)
40-49	0.20 (-0.38, 0.88)	0.04 (-0.08, 0.15)	0.12 (-0.45, 0.65)	0.50 (0.36, 0.65)	-0.02 (-0.50, 0.51)	0.06 (-0.05, 0.16)	0.27 (-0.31, 0.87)	0.12 (0.02, 0.22)
50-59	0.10 (-0.45, 0.69)	-0.04 (-0.15, 0.06)	0.23 (-0.35, 0.77)	0.64 (0.51, 0.76)	-0.11 (-0.62, 0.55)	0.03 (-0.10, 0.16)	0.08 (-0.58, 0.74)	-0.10 (-0.21, 0.02)
60-69	-0.13 (-0.64, 0.42)	-0.19 (-0.30, -0.09)	0.40 (-0.18, 0.86)	0.84 (0.72, 0.95)	-0.30 (-0.77, 0.29)	-0.06 (-0.18, 0.05)	-0.01 (-0.54, 0.50)	-0.12 (-0.25, 0.00)
70+	-0.71 (-1.21, -0.14)	-0.64 (-0.74, -0.54)	0.49 (0.07, 0.84)	1.11 (0.97, 1.25)	-0.39 (-0.79, 0.06)	-0.07 (-0.18, 0.03)	-0.04 (-0.42, 0.32)	-0.01 (-0.14, 0.11)
**Gender**								
Male	-0.10 (-0.68, 0.52)	-0.30 (-0.35, -0.25)	0.21 (-0.45, 0.73)	0.02 (-0.02, 0.06)	-0.11 (-0.59, 0.45)	0.07 (0.02, 0.12)	0.06 (-0.54, 0.61)	-0.24 (-0.30, -0.18)
Female	0.14 (-0.48, 0.80)	ref	0.12 (-0.51, 0.65)	ref	-0.22 (-0.72, 0.37)	ref	0.18 (-0.40, 0.76)	ref
**Race/ethnicity**								
Non-Hispanic Black	-0.08 (-0.68, 0.54)	-0.08 (-0.15, -0.01)	-0.01 (-0.66, 0.49)	-0.17 (-0.25, -0.08)	0.13 (-0.37, 0.74)	0.35 (0.28, 0.42)	0.04 (-0.57, 0.53)	-0.10 (-0.17, -0.03)
Mexican American	0.54 (-0.12, 1.19)	0.40 (0.31, 0.49)	0.13 (-0.66, 0.75)	0.04 (-0.08, 0.16)	-0.17 (-0.74, 0.43)	-0.05 (-0.18, 0.08)	-0.12 (-0.81, 0.47)	-0.23 (-0.35, -0.12)
Other Hispanic	0.36 (-0.28, 1.09)	0.33 (0.21, 0.45)	-0.13 (-0.78, 0.52)	-0.18 (-0.31, -0.04)	-0.19 (-0.72, 0.42)	-0.03 (-0.12, 0.07)	-0.07 (-0.71, 0.52)	-0.17 (-0.29, -0.05)
Asian	0.02 (-0.51, 0.62)	0.01 (-0.08, 0.09)	-0.08 (-0.78, 0.46)	-0.22 (-0.32, -0.12)	-0.12 (-0.55, 0.43)	0.06 (-0.02, 0.14)	0.28 (-0.33, 0.92)	-0.02 (-0.12, 0.07)
Other	0.00 (-0.69, 0.49)	-0.13 (-0.29, 0.04)	0.08 (-0.51, 0.53)	-0.07 (-0.22, 0.09)	-0.07 (-0.57, 0.55)	0.15 (0.00, 0.30)	0.05 (-0.42, 0.45)	-0.03 (-0.15, 0.09)
Non-Hispanic White	-0.03 (-0.63, 0.59)	ref	0.22 (-0.39, 0.73)	ref	-0.22 (-0.69, 0.36)	ref	0.16 (-0.41, 0.75)	ref
**Education**								
Less than high school	0.07 (-0.65, 0.86)	0.20 (0.12, 0.28)	0.19 (-0.49, 0.75)	0.12 (0.01, 0.23)	-0.22 (-0.74, 0.39)	0.07 (-0.03, 0.17)	-0.19 (-0.74, 0.34)	-0.38 (-0.47, -0.29)
High school graduate	0.07 (-0.59, 0.83)	0.15 (0.10, 0.21)	0.23 (-0.45, 0.80)	0.07 (-0.02, 0.16)	-0.11 (-0.65, 0.52)	0.12 (-0.01, 0.24)	0.01 (-0.60, 0.55)	-0.31 (-0.40, -0.22)
Some college	-0.01 (-0.65, 0.62)	-0.04 (-0.11, 0.03)	0.12 (-0.52, 0.64)	-0.02 (-0.09, 0.05)	-0.18 (-0.64, 0.40)	0.05 (-0.02, 0.12)	0.05 (-0.50, 0.61)	-0.25 (-0.36, -0.15)
College grad or higher	0.02 (-0.49, 0.54)	ref	0.13 (-0.46, 0.61)	ref	-0.16 (-0.62, 0.39)	ref	0.40 (-0.19, 0.99)	Ref
**Household income**								
<$20k	-0.13 (-0.88, 0.64)	-0.12 (-0.20, -0.04)	-0.03 (-0.71, 0.57)	-0.22 (-0.30, -0.13)	-0.25 (-0.72, 0.32)	0.05 (-0.05, 0.16)	-0.10 (-0.66, 0.35)	-0.29 (-0.39, -0.19)
$20k-$44.9k	-0.07 (-0.71, 0.70)	-0.02 (-0.09, 0.05)	0.15 (-0.57, 0.71)	-0.10 (-0.19, -0.01)	-0.20 (-0.72, 0.37)	0.02 (-0.07, 0.11)	-0.08 (-0.65, 0.43)	-0.30 (-0.37, -0.24)
$45k-$74.9k	0.11 (-0.50, 0.78)	0.07 (-0.02, 0.16)	0.23 (-0.37, 0.73)	-0.01 (-0.09, 0.08)	-0.13 (-0.63, 0.47)	0.03 (-0.05, 0.11)	0.07 (-0.45, 0.63)	-0.19 (-0.30, -0.08)
$75k+	0.06 (-0.45, 0.63)	ref	0.18 (-0.41, 0.68)	ref	-0.13 (-0.60, 0.46)	ref	0.40 (-0.23, 0.95)	ref
**Work status, last week**								
Did not work	-0.33 (-0.92, 0.35)	-0.32 (-0.42, -0.23)	0.14 (-0.52, 0.64)	-0.33 (-0.41, -0.26)	-0.35 (-0.8, 0.12)	-0.48 (-0.54, -0.42)	-0.04 (-0.53, 0.42)	-0.19 (-0.29, -0.09)
<40 hour	0.28 (-0.27, 0.92)	0.10 (0.01, 0.19)	0.09 (-0.56, 0.66)	-0.13 (-0.21, -0.06)	-0.25 (-0.77, 0.34)	-0.31 (-0.41, -0.22)	0.10 (-0.51, 0.76)	-0.14 (-0.25, -0.04)
40+ hour	0.18 (-0.37, 0.81)	ref	0.20 (-0.41, 0.74)	ref	0.08 (-0.43, 0.68)	ref	0.33 (-0.36, 0.93)	ref

*Abbreviations: CI* confidence interval, *HS* high school, *IQR* interquartile range, *NH* non-Hispanic, *PCA1-4* principal component analysis component 1-4

^a^ Derived from multiple linear regression models included all study characteristics and their categories simultaneously

Table 2 presents the association between participant characteristics and rest-activity profiles by weekday/weekends. PCA1-PCA3 showed weakened associations with work status in weekends than weekdays. In contrast, some results appeared stronger for weekends, including associations of a lower value of PCA1 among participants with lower income, a lower value of PCA2 among non-Hispanic Black participants, and a higher value of PCA3 among those in higher education and lower income groups. Finally, for PCA4, which likely presented two distinct patterns for weekdays and weekends, the previously noted associations between overall PCA4 and gender, education and income were only observed for weekdays, but not weekends. In contrast, a significantly lower weekend PCA4 or biphasic/late peak pattern was observed among non-Hispanic Black, other Hispanic and Asian groups.

**Table 2:** Associations (β (95% CI))^a^ between study characteristics and rest-activity profiles for weekdays and weekends separately in adults in the National Health and Nutrition Examination Survey (2011-2014)

	**PCA1 - high amplitude**		**PCA2 - early rise**		**PCA3 - prolonged activity window**		**PCA4 - biphasic pattern**	
	**Weekday**	**Weekend**	**Weekday**	**Weekend**	**Weekday**	**Weekend**	**Weekday**	**Weekend**
**Age**								
25-29	ref	ref	ref	ref	ref	ref	ref	ref
30-39	0.08 (-0.01, 0.18)	0.15 (0.04, 0.26)	0.16 (0.02, 0.29)	0.25 (0.12, 0.39)	0.09 (-0.02, 0.20)	0.04 (-0.08, 0.17)	0.03 (-0.10, 0.17)	0.06 (-0.08, 0.20)
40-49	0.07 (-0.04, 0.18)	0.05 (-0.07, 0.17)	0.47 (0.33, 0.61)	0.50 (0.33, 0.68)	0.11 (0.00, 0.21)	-0.11 (-0.23, 0.02)	0.02 (-0.09, 0.14)	0.06 (-0.08, 0.21)
50-59	-0.05 (-0.16, 0.06)	-0.01 (-0.15, 0.13)	0.61 (0.48, 0.73)	0.67 (0.51, 0.82)	0.05 (-0.08, 0.17)	-0.09 (-0.22, 0.05)	-0.20 (-0.32, -0.07)	0.14 (0.02, 0.27)
60-69	-0.17 (-0.26, -0.08)	-0.18 (-0.32, -0.04)	0.75 (0.62, 0.88)	0.88 (0.72, 1.03)	-0.09 (-0.20, 0.03)	-0.09 (-0.22, 0.04)	-0.17 (-0.31, -0.03)	0.17 (0.02, 0.32)
70+	-0.57 (-0.68, -0.47)	-0.64 (-0.77, -0.51)	1.06 (0.91, 1.20)	1.10 (0.95, 1.25)	-0.16 (-0.25, -0.06)	-0.04 (-0.17, 0.08)	-0.07 (-0.21, 0.07)	0.10 (-0.04, 0.24)
**Gender**								
Male	-0.26 (-0.31, -0.21)	-0.35 (-0.40, -0.29)	0.04 (0.00, 0.09)	0.02 (-0.03, 0.07)	-0.03 (-0.08, 0.02)	0.07 (0.02, 0.12)	-0.23 (-0.28, -0.18)	-0.04 (-0.11, 0.03)
Female	ref	ref	ref	ref	ref	ref	ref	ref
**Race/ethnicity**								
Non-Hispanic Black	-0.07 (-0.14, 0.00)	-0.11 (-0.18, -0.03)	-0.12 (-0.21, -0.03)	-0.21 (-0.31, -0.11)	0.29 (0.23, 0.35)	0.31 (0.22, 0.41)	-0.15 (-0.22, -0.08)	-0.23 (-0.29, -0.17)
Mexican American	0.43 (0.34, 0.53)	0.19 (0.11, 0.27)	0.03 (-0.10, 0.16)	0.02 (-0.12, 0.17)	-0.12 (-0.24, -0.01)	0.01 (-0.15, 0.17)	-0.15 (-0.27, -0.03)	-0.18 (-0.28, -0.07)
Other Hispanic	0.32 (0.22, 0.43)	0.22 (0.08, 0.37)	-0.18 (-0.31, -0.05)	-0.24 (-0.40, -0.07)	-0.08 (-0.19, 0.04)	0.08 (-0.03, 0.19)	-0.13 (-0.24, -0.01)	-0.23 (-0.34, -0.12)
Asian	0.01 (-0.08, 0.11)	0.00 (-0.09, 0.09)	-0.20 (-0.30, -0.11)	-0.18 (-0.28, -0.08)	0.03 (-0.06, 0.13)	0.20 (0.12, 0.29)	-0.03 (-0.13, 0.06)	-0.24 (-0.38, -0.10)
Other	-0.11 (-0.24, 0.03)	-0.11 (-0.31, 0.09)	-0.03 (-0.18, 0.13)	-0.13 (-0.29, 0.03)	0.09 (-0.07, 0.25)	0.13 (-0.07, 0.34)	-0.12 (-0.28, 0.03)	0.04 (-0.20, 0.28)
Non-Hispanic White	ref	ref	ref	ref	ref	ref	ref	ref
**Education**								
Less than high school	0.27 (0.20, 0.34)	0.06 (-0.01, 0.14)	0.12 (0.01, 0.23)	0.02 (-0.10, 0.15)	-0.04 (-0.14, 0.06)	0.08 (0.00, 0.17)	-0.40 (-0.51, -0.30)	0.05 (-0.05, 0.16)
High school graduate	0.21 (0.14, 0.27)	-0.03 (-0.10, 0.04)	0.06 (-0.03, 0.15)	-0.04 (-0.13, 0.05)	0.01 (-0.09, 0.11)	0.11 (0.00, 0.21)	-0.33 (-0.42, -0.23)	0.07 (-0.04, 0.18)
Some college	0.02 (-0.06, 0.09)	-0.14 (-0.22, -0.07)	-0.02 (-0.09, 0.06)	-0.07 (-0.14, 0.00)	-0.01 (-0.07, 0.06)	0.01 (-0.07, 0.10)	-0.25 (-0.35, -0.14)	-0.03 (-0.12, 0.07)
College grad or higher	ref	ref	ref	ref	ref	ref	ref	ref
**Household income**								
<$20k	-0.09 (-0.17, -0.01)	-0.15 (-0.23, -0.07)	-0.19 (-0.27, -0.10)	-0.21 (-0.28, -0.13)	-0.06 (-0.15, 0.03)	0.15 (0.03, 0.27)	-0.34 (-0.43, -0.25)	-0.05 (-0.14, 0.04)
$20k-$44.9k	0.03 (-0.04, 0.09)	-0.13 (-0.21, -0.06)	-0.08 (-0.18, 0.02)	-0.15 (-0.23, -0.07)	-0.08 (-0.17, 0.02)	0.12 (0.03, 0.21)	-0.31 (-0.40, -0.23)	-0.05 (-0.15, 0.05)
$45k-$74.9k	0.11 (0.04, 0.18)	0.01 (-0.09, 0.12)	-0.01 (-0.11, 0.08)	-0.02 (-0.10, 0.06)	-0.04 (-0.13, 0.04)	0.07 (-0.04, 0.18)	-0.22 (-0.32, -0.13)	0.07 (-0.03, 0.17)
$75k+	ref	ref	ref	ref	ref	ref	ref	ref
**Work status, last week**								
Did not work	-0.36 (-0.46, -0.26)	-0.25 (-0.33, -0.18)	-0.36 (-0.44, -0.29)	-0.14 (-0.23, -0.04)	-0.59 (-0.66, -0.52)	-0.08 (-0.15, -0.01)	-0.12 (-0.21, -0.02)	-0.11 (-0.19, -0.02)
<40 hour	0.05 (-0.03, 0.14)	0.12 (0.04, 0.20)	-0.15 (-0.23, -0.08)	0.00 (-0.09, 0.09)	-0.40 (-0.50, -0.30)	-0.01 (-0.12, 0.10)	-0.03 (-0.13, 0.07)	-0.13 (-0.27, 0.01)
40+ hour	ref	ref	ref	ref	ref	ref	ref	ref

*Abbreviations: CI* confidence interval, *HS* high school, *IQR* interquartile range, *NH* non-Hispanic, *PCA1-4* principal component analysis component 1-4

^a^ Derived from multiple linear regression models included all study characteristics and their categories simultaneously

Table 3 presents associations between rest-activity profiles and the odds of reporting poor or fair health, for overall and weekday/weekend profiles. Compared to participants with a higher eigenvalue for the high amplitude profile, those with a lower eigenvalue (i.e., participants with lower daily activity levels) were significantly more likely to report poor or fair health (OR _Q1 vs. Q5_ (95% CI), 2.61 (2.02, 3.37), *p-trend* < *0.0001*), and the results were similar for both weekday and weekend profiles. The early rise profile showed a suggestive inverse U-shaped association, with Q2 and Q3 each exhibiting a 28% increase in the OR for poor and fair health. Moreover, the results appeared more pronounced for the weekend early rise profile, showing a 43–44% increase. Participants in the lowest quintile of the eigenvalue for the prolonged activity window profile (i.e., shortest daytime active period) were 38% less likely to report poor or fair health, when compared to those in the highest quintile (0.62 (0.48, 0.82)). However, the association was weaker in the analysis focusing on weekday and weekend profiles specifically. Finally, participants in the lower quintiles (Q1-Q4) of the eigenvalues for the biphasic activity profile exhibited a 47%-62% increase in the odds of reporting poor or fair health when compared to those in the Q5, and the results were fairly consistent between weekday and weekend profiles.

**Table 3:** Associations ^a^ between overall, weekday and weekend rest-activity profiles and self-rated health ^b^ in adults in the National Health and Nutrition Examination Survey (2011-2014)

	**Self-rated health, poor or fair**					
	**Overall**		**Weekday**		**Weekend**	
	**No.**	**OR (95% CI)**	**No.**	**OR (95% CI)**	**No.**	**OR (95% CI)**
**fPCA1 - high amplitude**						
Q1 (low amplitude)	507	2.61 (2.02, 3.37)	485	2.62 (1.96, 3.49)	467	2.94 (2.26, 3.83)
Q2	357	1.99 (1.55, 2.55)	319	1.76 (1.33, 2.32)	367	2.37 (1.83, 3.05)
Q3	302	1.47 (1.16, 1.86)	291	1.52 (1.19, 1.94)	301	1.94 (1.51, 2.49)
Q4	280	1.29 (1.02, 1.63)	271	1.30 (1.00, 1.67)	264	1.55 (1.23, 1.95)
Q5 (high amplitude)	262	ref	247	ref	214	ref
*p-trend*		*<.0001*		*<.0001*		*<.0001*
**fPCA2 - early rise**						
Q1 (late rise)	317	1.07 (0.82, 1.39)	292	0.88 (0.65, 1.19)	274	1.07 (0.83, 1.38)
Q2	352	1.28 (1.00, 1.64)	333	1.01 (0.76, 1.33)	342	1.43 (1.12, 1.82)
Q3	349	1.28 (0.97, 1.70)	325	1.06 (0.78, 1.43)	350	1.44 (1.14, 1.83)
Q4	351	1.14 (0.92, 1.42)	327	1.00 (0.76, 1.33)	340	1.17 (0.96, 1.43)
Q5 (early rise)	339	ref	336	ref	307	ref
*p-trend*		*0.34*		*0.47*		*0.26*
**fPCA3 - prolonged activity window**						
Q1 (short activity window)	323	0.62 (0.48, 0.82)	327	0.80 (0.57, 1.12)	265	0.76 (0.55, 1.05)
Q2	341	0.83 (0.59, 1.16)	362	1.01 (0.75, 1.37)	349	1.13 (0.85, 1.49)
Q3	378	1.04 (0.74, 1.44)	368	1.19 (0.88, 1.59)	323	1.01 (0.71, 1.43)
Q4	345	0.84 (0.66, 1.05)	322	1.14 (0.83, 1.58)	341	0.95 (0.72, 1.25)
Q5 (long activity window)	321	ref	234	ref	335	ref
*p-trend*		*0.005*		*0.07*		*0.26*
**fPCA4 - biphasic pattern**						
Q1 (monophasic)	405	1.47 (1.11, 1.96)	383	1.54 (1.05, 2.27)	320	1.32 (1.02, 1.71)
Q2	396	1.50 (1.07, 2.10)	394	1.69 (1.30, 2.18)	359	1.47 (1.11, 1.94)
Q3	384	1.62 (1.22, 2.17)	362	1.58 (1.11, 2.25)	354	1.47 (1.13, 1.91)
Q4	330	1.56 (1.18, 2.07)	298	1.38 (1.00, 1.90)	308	1.19 (0.90, 1.56)
Q5 (biphasic)	193	ref	176	ref	272	ref
*p-trend*		*0.05*		*0.009*		*0.01*

*Abbreviations: CI* confidence interval, *HS* high school, *IQR* interquartile range, *NH* non-Hispanic, *PCA1-4* principal component analysis component 1-4

^a^ adjusted for age (25-29, 30-39, 40-49, 50-59, 60-69, and 70+), gender (male, female), race/ethnicity (non-Hispanic White, non-Hispanic Black, Mexican American, Other Hispanic, Asian, and other), education (less than high school, high school graduate, some college, college graduate or higher), household income (<$20k, $20k-$44.9k, $45k-$74.9k, ≥$75k), work status in the past week (did not work, worked for <40 hours, worked 40 hours or more)

^b^included in the model as a dichotomous outcome, indicating if the participant reported poor or fair health status

## Discussion

In a nationally representative sample of US adults, we identified four distinct profiles for the 24-h rest-activity cycle. We found considerable variation in these profiles across different subgroups by age, gender, race/ethnicity, SES and work status. We also observed associations between rest-activity profiles and self-rated health status.

### The high amplitude profile

The High amplitude profile (PCA1) appeared to be primarily driven by a daytime physical activity levels. Previous studies using fPCA also found the high amplitude profile as the primary component substantially explaining the population variance [12–14, 16], varying from 34.3% in a study of 359 adults in the Netherlands [16] and 50% in 2,796 men in the Osteoporotic Fractures in Men (MrOS) study in the US [14]. We found a high correlation between the weekday and weekend high amplitude profiles, suggesting a relatively high consistency in physical activity levels throughout the week among individuals. The strong inverse association between high amplitude and the odds or reporting fair or poor health was also consistent with the well-documented association between health status and physical activity levels. Our findings and those from previous studies suggest that overall daytime physical activity level represents a primary, possibly most distinguishing feature for rest-activity patterns.

The high amplitude profile was significantly associated with almost every participant characteristic in our study, although some findings were consistent with previous literature while others not. For example, lower amplitude was positively associated with age, which was consistent with the well-established evidence suggesting a decline in physical activity levels in older adults [24, 25]. In addition, relative to their non-Hispanic white counterparts, Hispanic participants exhibited a higher amplitude while non-Hispanic black participants exhibited a lower amplitude, which agreed with previously reported racial/ethnic differences in physical activity [26]. For gender, we found a lower amplitude in men, similar to previous studies that reported lower levels of total activity measured by wrist actigraphy in men than in women [27, 28]. Notably, an analysis using hip-worn accelerometry data in NHANES 2003–2006 reported *higher*activity levels in men [26]. The discrepancy may be explained by the higher level of women’s engagement in light-intensity household activities, which require more upper-body movement and thus are better captured by wrist-worn actigraphy [29].

High amplitude profile was positively associated with income and the association was more pronounced on weekends than on weekdays. A previous study in NAHNES 2003–2006 also reported that higher income was associated with higher activity levels, especially less frequent but higher intensity physical activity on weekends [30], suggesting that the association with income may be explained by weekend recreational/exercise activities. High amplitude profile, on the other hand, was negatively associated with education and only for the weekdays, suggesting that this association may be driven by weekday occupational activities, possibly because the lower education groups tend to hold labor-intensive jobs. Moreover, we observed that participants who did not work in the previous week had a substantially lower amplitude compared to those working 40 h or more, again supporting occupational activity as a key contributor to the daily physical activity, as reported in previous research [31].

### The early rise profile

The early rise profile (PCA2) captures the timing aspect of the rest-activity cycle. All previous fPCA studies identified at least one component related to early vs. late activity window, explaining 11.4%-23.0% of total activity variation [12–14, 16]. We found a relatively high weekday-weekend correlation (0.65) for the early rise profile, which supports an important role of intrinsic factors in regulating the timing of diurnal behaviors. On the other hand, the imperfect correlation and weekday-weekend difference in the association between the early rise profile and sociodemographic factors, as described below, also suggested a considerable impact of environmental factors, such as work schedule, on the timing of sleep. A growing body of literature has linked the evening chronotype (i.e., the preference to sleep at a later time) with disease risks such as mental disorders and metabolic dysfunction [32, 33]. However, in our analysis, we did not observe a relationship between a lower eigenvalue for the early-rise profile and higher odds of reporting poor or fair health. The discrepancy between our findings and those in earlier research may suggest that although correlated with sleep timing, early rise profile is not redundant with the measure of chronotype, and its relationship with health should be examined in future research.

The strong positive relationship between age and the early rise profile observed in our study has been consistently reported previously [34, 35]. There is a well-documented developmental shift of the human circadian clocks towards a delayed phase during adolescence and early adulthood, and after which the rhythm starts to become more advanced as people age [36]. Gender differences in chronotype has also been well documented where women generally have a more advanced phase [34, 35], possibly driven by a shorter intrinsic circadian period among women [37]. However, such gender difference was not observed in our analysis either in overall or weekday- and weekend-specific analysis. Moreover, two previous studies also did not find a significant gender difference in either acrophase or the early rise profile [15, 16]. It is worth noting that studies reporting no gender differences tended to focus on activity timing, not chronotype or sleep timing. It is possible that chronotype and early rise profile, although likely correlated, represent two distinct measures that may be influenced by different internal and external factors. Racial/ethnic differences reported in earlier studies were almost exclusively made on the Black-White comparisons, and the results were mixed, with several studies reporting an earlier chronotype among Blacks [38–40], while others reporting no racial difference [15, 41]Hardly any research reported chronotype or rest-activity timing among Hispanic and Asian populations. We filled the knowledge gap by using a diverse sample representing the US population, and reported that except for Mexican Americans, all other non-White racial/ethnic groups exhibited a lower eigenvalue for the early rise profile, a novel finding that needs to be confirmed by future research.

Previous studies on the relationship between SES (e.g., education and income) and timing of rest-activity patterns were inconclusive: Mitchell et al. reported an association between earlier acrophase and lower education, but no difference across income groups, [15] while Difrancesco et al. found no relationship between early vs. late activity profiles and education. [16] Limited research on SES and the rest-activity timing and the mixed findings therein warrant future investigations to confirm our findings. In addition, we found a significant relationship between not working/working for < 40 h and a later rise time, particularly on weekdays, suggesting the important role of work schedule in determining rest-activity patterns in the working population.

### The prolonged activity window profile

The prolonged activity window profile is characterized by an early rise time but also a later time in the declining phase of the rest-activity pattern, resulting in a longer day activity window, and a similar profile was reported in previous studies in the MrOS [14] and a sample of Dutch adults [16]. We found higher odds of reporting fair or poor health was associated with prolonged activity window, as opposed to shorter window. A longer activity window may reflect shorter, less regular, or less restful sleep, and thus the observed association with self-rated health may be driven by well-established adverse health effects of sleep deficiency [42].

We found Black participants were more prone to exhibit the prolonged activity window profile, consistent with the current literature invariantly reported that Black Americans had shorter sleep duration than their White counterparts [43]. We also found a strong relationship between not working or working for < 40 h and a lower eigenvalue for this profile, particularly on weekdays, which suggests a longer weekday sleep duration among these groups and supports the crucial role of work on shaping sleep and rest-activity patterns. Finally, we found that people with lower education or income were more likely to exhibit prolonged activity window profile, but only on weekends and not weekdays, suggesting a comparatively shorter sleep duration on weekends among these groups. It is noteworthy that this finding may be driven by a longer catch-up sleep on weekends among higher SES groups. Several studies reported that weekend catch-up sleep may be associated with health benefits, particularly among short sleepers [44–46]. Thus the inability to have catch-up sleep on weekends among low SES groups may be a public health concern.

### The biphasic pattern profile

The biphasic profile was reported by previous fPCA studies [12–14, 16], and also by several recent papers focusing on sedentary behaviors and moderate-to-vigorous physical activity [31, 47]. Although this profile does not explain the rest-activity pattern nearly as much as, say, the amplitude profile, its constant appearance in various populations suggests it as an important feature of the rest-activity cycle. In our analysis, participants exhibiting less biphasic pattern were more likely to report fair or poor health, contrary to the finding from MrOS study where biphasic pattern was associated with higher cardiovascular-related mortality in older men [14]. Notably, the biphasic pattern in the MrOS analysis was inseparable from a longer activity window, while in our study these profiles emerged as two unique and distinguishable components. The disagreements in findings may be attributable to different health outcomes assessed in the two studies, and/or different age, gender and racial/ethnic compositions in the two study populations, which led to different rest-activity profiles generated by fPCA. More research is needed to clarify the health effect of the biphasic pattern.

The biphasic profile was more pronounced among participants with higher education and income, and who reported working 40 h or more. Moreover, the biphasic pattern was only present on weekdays, suggesting a role of occupational factors. Therefore, the morning and evening peaks of activity in this profile may correspond to work-related commute and social and family activities before and after work hours [31, 47], while the reduced activity during the middle day may correspond to a more sedentary 9–5 work period typical of white-collar jobs. We also found that the biphasic pattern was more pronounced in women than in men, which may be explained by women’s higher engagement in household chores and family obligations before and after work. Finally, the biphasic pattern was less obvious in non-Hispanic Black and Hispanic groups, which may be partially explained by racial/ethnic differences in occupations.

### Strengths and limitations

A major strength of our study is its large, diverse and nationally representative sample, which allowed us to evaluate rest-activity patterns in the general US population and make comparisons across subgroups by demographic, socioeconomic and work characteristics. Moreover, we took a flexible, data-driven approach without a priori assumptions to characterize rest-activity patterns, and derived multi-dimensional features of the 24-h movement behaviors. Finally, we performed stratified analysis by weekdays/weekends, allowing for the assessment of potential impact of workday schedule on the rest-activity cycles.

Our study also has several limitations. First, although fPCA is an innovative method with many advantages, it also has some limitations. Most notably, the derived profiles are sample-specific and may not be applicable to other populations. It is thus important to include a large, diverse and representative sample such as the NHANES to improve the generalizability of study findings. Moreover, the method can be subject to over-fitting, and profiles obtained can be difficult to interpret. However, we did identify distinct and interpretable profiles in our sample. Second, we only examined six participant characteristics, while many other factors such as lifestyle, health conditions, and social interactions may play a role in shaping rest-activity behaviors. Third, the work variable in our study was crude and only collected information on the work status in the previous week. Given our finding of an important role of work status, future studies should include more detailed information and long-term assessment to better examine employment and occupational factors in relation to rest-activity patterns. Fourth, despite the representativeness of our study sample, the profiles and associations reported by our study may not be generalizable to other samples, especially populations with highly distinct rest-activity patterns such as night-shift workers and severely ill patients. Fifth, we focused on self-rated health as a general measure of health, whereas rest-activity profiles may have different associations with different disease outcomes. Finally, NHANES is a cross-sectional study, and therefore we could not determine the direction of certain associations, particularly those related to SES, work status, and self-rated health.

## Conclusions

Our study identified distinct rest-activity profiles among the US population and provided supporting evidence that human rest-activity patterns are shaped by demographic, socioeconomic and work factors. Some of the results, especially those for the high amplitude component, were consistent with previous studies focusing on individual behavior patterns such as overall physical activity. However, we also identified and examined profiles that captured additional aspects of the behavior (e.g., timing, biphasic shape) of the diurnal rest-activity patterns, for which there has been limited study on their sociodemographic, occupational and health correlates. Currently, behavioral interventions largely focus on altering individual behaviors, such as increasing physical activity levels, reducing prolonged sitting, and improving sleep quality. More recently, researchers in the field of circadian physiology have also developed strategies that may help improving the rhythmicity of diurnal behaviors, such as timed melatonin treatment, light exposure, and exercise regimes [48, 49]. We believe individuals may benefit from multiple types of interventions, and a comprehensive characterization of various aspects of the rest-activity pattern using algorithms such as the fPCA may help identifying areas for behavioral improvement and evaluating the efficacy of interventions.

Overall, the relationships between environmental exposures and behavior patterns are complicated, and may also intertwine with the internal circadian clock. We encourage future studies to focus on identifying additional individual and environmental factors that explain rest-activity patterns, and.

## Supplementary Information

**Additional file 1: Supplementary Figure 1. **Weekday rest-activity profiles of 24-hour actigraphy data from adults in the National Health and Nutrition Examination Survey (2011-2014). Each panel depicts the mean 24-hour activity patterns for participants with high (solid line) and low (dotted) eigenvalues of the first four components derived from the functional principal component analysis (PCA): A) The first component (48.5% variance), with higher eigenvalues representing a higher amplitude; B) the second component (20.6% variance), with higher eigenvalues representing earlier rise time; C) the third component (10.1% variance), with higher eigenvalues representing a more prolonged daytime activity window; D) the forth component (6.2% variance), with higher eigenvalues representing a more pronounce biphasic pattern characterized by a mid-day dip in activity. **Supplementary Figure 2. **Weekend rest-activity profiles of 24-hour actigraphy data from adults in the National Health and Nutrition Examination Survey (2011-2014). Each panel depicts the mean 24-hour activity patterns for participants with high (solid line) and low (dotted) eigenvalues of the first four components derived from the functional principal component analysis (PCA): A) The first component (42.4% variance), with higher eigenvalues representing a higher amplitude; B) the second component (21.8% variance), with higher eigenvalues representing earlier rise time; C) the third component (10.1% variance), with higher eigenvalues representing a more prolonged daytime activity window; D) the forth component (6.7% variance), with higher eigenvalues representing a later activity peak. **Supplementary Table 1.** Distribution of study characteristics among 7,657 participants aged 25 or older in the National Health and Nutrition Examination Survey (2011-2014). **Supplementary Table 2.** Pearson correlation coefficients between overall, weekday and weekend rest-activity profiles among adults in the National Health and Nutrition Examination Survey (2011-2014). 

## Data Availability

All NHANES datasets used in the current analysis are available for download from the NHANES website.

## Abbreviations

MrOS: Osteoporotic Fractures in Men study
NHANES: National Health and Nutrition Examination Survey
PCA: Principal component analysis component 1–4
SES: Socioeconomic status
