# Supplementary material for: Rest-activity profiles among U.S. adults in a nationally representative sample: a functional principal component analysis
Source: Int J Behav Nutr Phys Act. 2022 Mar 24;19:32. doi: 10.1186/s12966-022-01274-4 (PMC8944104; doi:10.1186/s12966-022-01274-4)
Supplement: Supplementary file 1 — Additional file 1: Supplementary Figure 1. Weekday rest-activity profiles of 24-hour actigraphy data from adults in the National Health and Nutrition Examination Survey (2011-2014). Each panel depicts the mean 24-hour activity patterns for participants with high (solid line) and low (dotted) eigenvalues of the first four components derived from the functional principal component analysis (PCA): A) The first component (48.5% variance), with higher eigenvalues representing a higher amplitude; B) the second component (20.6% variance), with higher eigenvalues representing earlier rise time; C) the third component (10.1% variance), with higher eigenvalues representing a more prolonged daytime activity window; D) the forth component (6.2% variance), with higher eigenvalues representing a more pronounce biphasic pattern characterized by a mid-day dip in activity. Supplementary Figure 2. Weekend rest-activity profiles of 24-hour actigraphy data from adults in the National Health and Nutrition Examination Survey (2011-2014). Each panel depicts the mean 24-hour activity patterns for participants with high (solid line) and low (dotted) eigenvalues of the first four components derived from the functional principal component analysis (PCA): A) The first component (42.4% variance), with higher eigenvalues representing a higher amplitude; B) the second component (21.8% variance), with higher eigenvalues representing earlier rise time; C) the third component (10.1% variance), with higher eigenvalues representing a more prolonged daytime activity window; D) the forth component (6.7% variance), with higher eigenvalues representing a later activity peak. Supplementary Table 1. Distribution of study characteristics among 7,657 participants aged 25 or older in the National Health and Nutrition Examination Survey (2011-2014). Supplementary Table 2. Pearson correlation coefficients between overall, weekday and weekend rest-activity profiles among adults in the National Health and Nutritio [file 12966_2022_1274_MOESM1_ESM.docx]

A) High amplitude (PCA1)

B) Early rise (PCA2)

C) Prolonged activity (PCA3)

D) Biphasic pattern (PCA4)

**Supplementary Figure 1** Weekday rest-activity profiles of 24-hour actigraphy data from adults in the National Health and Nutrition Examination Survey (2011-2014). Each panel depicts the mean 24-hour activity patterns for participants with high (solid line) and low (dotted) eigenvalues of the first four components derived from the functional principal component analysis (PCA): A) The first component (48.5% variance), with higher eigenvalues representing a higher amplitude; B) the second component (20.6% variance), with higher eigenvalues representing earlier rise time; C) the third component (10.1% variance), with higher eigenvalues representing a more prolonged daytime activity window; D) the forth component (6.2% variance), with higher eigenvalues representing a more pronounce biphasic pattern characterized by a mid-day dip in activity.

A) High amplitude (PCA1)

B) Early rise (PCA2)

C) Prolonged activity (PCA3)

D) Late peak/Biphasic pattern (PCA4)

**Supplementary Figure 2** Weekend rest-activity profiles of 24-hour actigraphy data from adults in the National Health and Nutrition Examination Survey (2011-2014). Each panel depicts the mean 24-hour activity patterns for participants with high (solid line) and low (dotted) eigenvalues of the first four components derived from the functional principal component analysis (PCA): A) The first component (42.4% variance), with higher eigenvalues representing a higher amplitude; B) the second component (21.8% variance), with higher eigenvalues representing earlier rise time; C) the third component (10.1% variance), with higher eigenvalues representing a more prolonged daytime activity window; D) the forth component (6.7% variance), with higher eigenvalues representing a later activity peak.

**Supplementary Table 1** Distribution of study characteristics among 7,657 participants aged 25 or older in the National Health and Nutrition Examination Survey (2011-2014).

|  | **N (%) ^a^** |
| --- | --- |
| **Age** |  |
| 25-29 | 634 (9.1) |
| 30-39 | 1451 (19.1) |
| 40-49 | 1440 (20.5) |
| 50-59 | 1371 (20.9) |
| 60-69 | 1420 (16.4) |
| 70+ | 1341 (13.9) |
| **Gender** |  |
| Male | 3632 (47.2) |
| Female | 4025 (52.8) |
| **Race/ethnicity** |  |
| Non-Hispanic Black | 1747 (10.6) |
| Mexican American | 853 (7.7) |
| Other Hispanic | 696 (5.4) |
| Asian | 865 (4.6) |
| Other | 205 (2.5) |
| Non-Hispanic White | 3291 (69.1) |
| **Education** |  |
| Less than high school | 1680 (15.4) |
| High school graduate | 1660 (20.8) |
| Some college | 2244 (31.1) |
| College grad or higher | 2073 (32.7) |
| **Household income** |  |
| <$20k | 1779 (15.4) |
| $20k-$44.9k | 2293 (26.0) |
| $45k-$74.9k | 1410 (20.8) |
| $75k+ | 2175 (37.8) |
| **Work status, last week** |  |
| Did not work | 3690 (41.2) |
| <40 hour | 1222 (17.4) |
| 40+ hour | 2745 (41.4) |
| **Self-rated health** |  |
| Excellent | 654 (10.0) |
| Very good | 1914 (30.2) |
| Good | 2853 (37.2) |
| Fair | 1418 (14.2) |
| Poor | 290 (2.7) |
| Missing | 528 (5.7) |

^a^ survey weight was applied in calculating %.

**Supplementary Table 2** Pearson correlation coefficients between overall, weekday and weekend rest-activity profiles among adults in the National Health and Nutrition Examination Survey (2011-2014).

|  |  | **PCA1 - high amplitude** | | | **PCA2 - early rise** | | | **PCA3 - prolonged activity** | | | **PCA4 - biphasic pattern** | | |
| --- | --- | --- | --- | --- | --- | --- | --- | --- | --- | --- | --- | --- | --- |
|  |  | **Overall** | **Weekday** | **Weekend** | **Overall** | **Weekday** | **Weekend** | **Overall** | **Weekday** | **Weekend** | **Overall** | **Weekday** | **Weekend** |
| **PCA1 - high amplitude** | **Overall** | 1.00 | 0.97 | 0.86 | 0.00 | -0.08 | 0.02 | 0.00 | 0.02 | 0.04 | 0.00 | 0.04 | 0.01 |
|  | **Weekday** |  | 1.00 | 0.72 | 0.07 | 0.00 | 0.04 | 0.00 | 0.00 | 0.03 | -0.04 | 0.00 | 0.02 |
|  | **Weekend** |  |  | 1.00 | -0.11 | -0.17 | 0.00 | -0.03 | 0.05 | 0.00 | 0.10 | 0.13 | 0.00 |
| **PCA2 - early rise** | **Overall** |  |  |  | 1.00 | 0.96 | 0.82 | 0.00 | -0.07 | -0.06 | 0.00 | 0.06 | 0.11 |
|  | **Weekday** |  |  |  |  | 1.00 | 0.65 | 0.07 | 0.00 | -0.06 | -0.03 | 0.00 | 0.11 |
|  | **Weekend** |  |  |  |  |  | 1.00 | -0.06 | -0.09 | 0.00 | 0.06 | 0.11 | 0.00 |
| **PCA3 - prolonged activity** | **Overall** |  |  |  |  |  |  | 1.00 | 0.91 | 0.67 | 0.00 | -0.22 | 0.00 |
|  | **Weekday** |  |  |  |  |  |  |  | 1.00 | 0.40 | 0.22 | 0.00 | 0.00 |
|  | **Weekend** |  |  |  |  |  |  |  |  | 1.00 | -0.02 | -0.20 | 0.00 |
| **PCA4 - biphasic pattern** | **Overall** |  |  |  |  |  |  |  |  |  | 1.00 | 0.91 | 0.31 |
|  | **Weekday** |  |  |  |  |  |  |  |  |  |  | 1.00 | 0.14 |
|  | **Weekend** |  |  |  |  |  |  |  |  |  |  |  | 1.00 |
